# The Association Between Neutrophil-to-Lymphocyte Ratio, Atherogenic Index of Plasma, and Cardiovascular Disease Incidence

**DOI:** 10.1155/mi/3302911

**Published:** 2025-08-22

**Authors:** Hongxin Cheng, Wen Zhong, Hanbin Li, Lu Wang, Chengqi He, Liyi Huang, Chenying Fu, Quan Wei

**Affiliations:** ^1^Department of Rehabilitation Medicine Center and Institute of Rehabilitation Medicine, West China Hospital, Sichuan University, Chengdu, Sichuan, China; ^2^Key Laboratory of Rehabilitation Medicine in Sichuan Province, Chengdu, Sichuan, China; ^3^State Key Laboratory of Biotherapy and National Clinical Research Center for Geriatrics, West China Hospital, Sichuan University, Chengdu, Sichuan, China; ^4^Geriatric Health Care and Medical Research Center, National Clinical Research Center for Geriatrics, West China Hospital, Sichuan University, Chengdu, Sichuan, China

**Keywords:** atherogenic index of plasma (AIP), cardiovascular diseases (CVDs), neutrophil-to-lymphocyte ratio (NLR)

## Abstract

**Background:** With the incidence of cardiovascular disease (CVD) escalating annually, the significance of investigating the correlation between inflammatory markers and lipid-related indices, notably the neutrophil-to-lymphocyte ratio (NLR) and atherogenic index of plasma (AIP), is increasingly paramount. This study aimed to elucidate the distinct and combined roles of NLR and AIP concerning the incidence of CVD.

**Methods:** Diagnoses of CVD were established based on self-reported standardized medical condition questionnaires from participants. NLR was computed by dividing the peripheral blood neutrophil count by the lymphocyte count. AIP was calculated as log [triglyceride (TG, mg/dL)/high-density lipoprotein cholesterol (HDL-C, mg/dL)]. The study's primary outcome was the incidence of CVD. To ensure the reliability and accuracy of the results, the analysis incorporated sample weights and complex survey designs.

**Results:** The final analysis included 13,184 individuals. Higher levels of NLR and AIP were independently associated with CVD. After adjusting for all variables, compared to Q1 of AIP or NLR, Q4 of AIP (OR 1.54, 95% CI: 1.19–1.98) and NLR (OR 1.54, 95% CI: 1.19–1.98) was significantly associated with CVD. The joint effects showed that participants with higher levels of NLR and AIP had a significantly higher OR of 1.41(1.06, 1.87). The combination of NLR and AIP has better predictive efficacy (AUC: 0.629) for CVD than alone.

**Conclusion:** This cohort suggests combined effects between the NLR and AIP on CVD. Our findings provide clinical implications for monitoring and managing NLR and AIP levels to mitigate the development of CVD.

## 1. Background

Globally, cardiovascular diseases (CVDs) are the leading cause of human mortality. According to statistics, the number of deaths caused by CVD reached 20.5 million in 2021 [1], accounting for approximately one-third of the total global deaths. Among these CVD, ischemic heart disease (9.1 million deaths), and stroke (6.6 million deaths) accounted for as much as 85% of the deaths [2]. Notably, with the intensifying trend of global population aging, the number of deaths from CVD has continued to increase over the past 30 years [3], exacerbating the pressure on public health. The high incidence and mortality of CVD make them a major issue in global public health. Therefore, identifying risk factors for CVD occurrence is of paramount importance for guiding preventive measures and developing strategies to alleviate the burden of this condition on both individuals and healthcare systems worldwide.

CVD pathogenesis involves complex interplay between chronic inflammation and dyslipidemia [4]. Consequently, there is growing interest in developing integrated biomarkers that capture both processes for improved risk stratification [5]. Individual inflammatory indices, such as the neutrophil-to-lymphocyte ratio (NLR), systemic immune-inflammation index (SII), and lymphocyte-to-monocyte ratio (LMR), have demonstrated predictive value in CVD [6–10]. Similarly, lipid-centric indices like the atherogenic index of plasma (AIP) and the triglyceride-glucose (TyG) index are strongly associated with cardiovascular risk [7, 11]. Recent studies have begun exploring combinations of inflammatory and lipid markers, for example, SII and TyG, suggesting they have a clear positive correlation in CVD [7]. Compared to TyG index or NLR, the combination of the TyG index and NLR is beneficial to improve the diagnostic accuracy of coronary artery disease and coronary artery disease severity [12]. However, despite the established roles of NLR and AIP in CVD, their joint effect remains uninvestigated. Among these indicators, NLR, a straightforward and easily calculated indicator, is determined by dividing the neutrophil count (count/ml) by the lymphocyte count (count/mL) [13]. This ratio serves as a comprehensive measure of both the natural (neutrophils) and acquired (lymphocytes) immune responses. Neutrophils, serving as the body's primary line of defense, can swiftly respond to infections or inflammations via mechanisms, such as chemotaxis, phagocytosis, and cytokine release [14]. On the other hand, lymphocytes, a crucial component of the immune system, possess the ability to identify and combat pathogens [15]. An abnormally elevated or reduced NLR value might signify an overactive or inadequate inflammatory response, or a dysfunctional or excessively strong immune response, potentially resulting in various health complications. Research has established a close association between NLR and a range of diseases, including CVD [6, 16], autoimmune diseases [17], infectious diseases [18], malignant tumors [19], and diabetes [20]. Although NLR is a powerful marker associated with disease severity and mortality, past studies have rarely explored its relationship with the incidence of CVD in the general population.

In recent years, AIP, defined as the logarithmic ratio of triglycerides (TGs) to high-density lipoprotein cholesterol (HDL-C), has emerged as a novel, cost-effective lipid biomarker that reflects atherogenic dyslipidemia with superior sensitivity and specificity compared to traditional lipid markers, such as TG and low-density lipoprotein cholesterol (LDL-C) [21, 22]. Studies have identified AIP as an independent biomarker for atherosclerosis [23], given its strong association with the size and density of lipoprotein particles and the peroxide production rate [24]. Moreover, AIP has been linked to CVD risk in cohort studies, predominantly involving Asian populations, yet the broader applicability of these findings to diverse populations remains to be confirmed [11, 25, 26]. While most research has emphasized the association between AIP and disease risk, limited attention has been given to understanding its relationship with the incidence of CVD. Therefore, further studies are essential to elucidate the potential of AIP as a reliable indicator for assessing the risk of CVD occurrence.

Accordingly, the objective of this study was to leverage data from the National Health and Nutrition Examination Survey (NHANES) to examine the correlation between NLR, AIP, and the incidence of CVD in the general population.

## 2. Methods

### 2.1. Study Population

All data for this study are sourced from NHANES database, freely available on its official website [27, 28]. This database, led by the U.S. Centers for Disease Control and Prevention, is based on a representative sample survey conducted biennially in the United States since 1999. The NHANES survey is designed to collect comprehensive data on the diet, nutritional status, and chronic disease prevalence among the U.S. population. Approximately 5000 participants are surveyed each year, and the data is publicly published every 2 years. The data collection process has been approved by the appropriate research ethics committee, and all participants have provided written informed consent.

### 2.2. Calculation of the NLR and AIP

The NLR is determined by dividing the absolute count of neutrophils in peripheral blood by the absolute count of lymphocytes [29]. AIP is calculated using the formula log [TG (mg/dL)/HDL-C (mg/dL)], where TGs represents triglycerides and HDL-C denotes high-density lipoprotein cholesterol [30].

These indexes, along with other blood-related parameters, is derived from the laboratory test data section of the NHANES database. For the purpose of this project, participants are initially subjected to physical examinations and laboratory tests. The process of collecting laboratory test data involves the examination and collection of laboratory blood samples, a task meticulously carried out by trained medical personnel.

### 2.3. Definition of CVD

Our primary outcome is cardiovascular morbidity. The diagnosis of CVD is based on the self-reported standardized medical condition questionnaire from participants in the NHANSE database [31]. During the interview process, participants were asked the following questions: “Has a doctor or other health professional told you that you have congestive heart failure?", “Has a doctor or other health professional told you that you have coronary heart disease?", “Has a doctor or other health professional told you that you have angina?", “Has a doctor or other health professional told you that you have had a heart attack?”, or “Has a doctor or other health professional told you that you have had a stroke?” If the participant's answer to any of the above questions is affirmative, they are considered to have CVD.

### 2.4. Covariates Collection

The variables of the included population are derived from the NHANES database and encompass four main categories: Basic demographic information: This includes age, gender, race, education, and family income-poverty ratio. Physical examination: variables include body mass index (BMI), waist circumference (WC), systolic blood pressure (SBP), and diastolic blood pressure (DBP); Laboratory tests: This includes urine creatinine, total cholesterol (TC), HDL-C, TGs, LDL-C, fasting blood glucose (FBG), glycated hemoglobin (HbA1c%), platelet count, and lymphocyte and neutrophil count; Questionnaire data: This includes smoking status, drinking status, and history of diabetes, hypertension.

To facilitate a more intuitive and clear observation of the population characteristics, we adjusted some continuous variables to categorical variables. For instance, age is categorized into two groups: more than 65 years and less than 65 years old. Ethnicity is divided into Mexican American, other Hispanic, Non-Hispanic White, Non-Hispanic Black, and other/multiracial. Education attainment is divided into less than 9th grade, 9–11th grade, high school grad/ged, some college or aa degree, and college graduate or above. Based on the patient's drinking questionnaire, we classified the patient's drinking habits and frequency as nondrinker, 1–5 drinks/month, 5–10 drinks/month, and 10+ drinks/month. Smoking status is defined as a never smoker (smokes less than 100 cigarets in a lifetime), a former smoker (smoked more than 100 cigarets but does not smoke at all now), and a current smoker (smoked more than 100 cigarets and now smokes daily or regularly). And we defined hypertension as follows: (1) Participants who have been told by a physician or other health professional to have hypertension and who are or have taken hypertension medications to lower their blood pressure are considered to be hypertensive, regardless of their current blood pressure values; (2) Other participants were divided into a nonhypertension group (SBP < 140 mmHg and DBP < 90 mmHg) and a hypertension group (SBP ≥ 140 mmHg and DBP ≥ 90 mmHg). Diabetes mellitus was defined as follows: (1) Participants who have been told by a physician or other health professional to have diabetes and are now taking insulin or have taken diabetes medications are considered to be diabetic, regardless of their current blood glucose levels; (2) Participants with a FBG concentration ≥ 126 mg/dL or HbA1*c* ≥ 6.5% are also considered diabetic.

### 2.5. Statistical Analysis

Following the NHANES analysis standards, we factored in sample weights and complex survey designs during the analysis to ensure the accuracy and reliability of the results. Sample-specific weights account for the unequal probabilities of individual sampling, while complex survey designs encompass multi-stage probability sampling and oversampling. All analyses were conducted under complex weighting, with the sample weights calculated as 2/10 of the 4-year weight from 1999 to 2002 and 1/10 of the 2-year weight from 2003 to 2018. Continuous variables were expressed as weighted means (standard deviation) based on the complex survey design. Group differences were evaluated using statistical tests appropriate for complex sampling, such as *t*-tests or rank-sum tests where applicable. Categorical variables were summarized as unweighted frequencies (*n*) and weighted percentages (%). Rao and Scott's second-order correction for the chi-squared test was applied to compare differences among categorical variables. All data analyses accounted for the complex survey design, including stratification, clustering, and sample weights, as implemented in the NHANES dataset processing.

Both NLR and AIP are continuous variables and were categorized into two levels: high and low, based on their respective weighted median values calculated using the complex survey design. This classification resulted in four distinct groups: Group 1, representing individuals with low NLR and low AIP values; Group 2, with high NLR and low AIP values; Group 3, with low NLR and high AIP values; and Group 4, with high NLR and high AIP values. Furthermore, the variables AIP and NLR were categorized into quartiles by calculating weighted quantiles at the 0th, 25th, 50th, 75th, and 100th percentiles. These quantiles were then used as thresholds to divide each variable into four quartiles (Q1 to Q4), reflecting progressively higher levels of AIP and NLR.

To investigate the relationship between NLR, AIP, and CVD incidence in these groups, we initially employed a weighted logistic regression model analysis. Model I is unadjusted, while Model II is adjusted for age, gender, ethnicity, education, and family income–poverty ratio. Model III is adjusted for age, gender, race, education, family income–poverty ratio, BMI, WC, SBP, DBP, urine creatinine, FBG, HbA1c%, TC, TG, HDL-C, LDL-C, history of diabetes, history of hypertension, and history of coronary heart disease. We compute the odds ratio (OR) and the 95% confidence interval (CI). Subgroup analyses are conducted controlling for variables in Model III, primarily focusing on gender, age, drinking status, smoking status, BMI, presence of diabetes, and hypertension.

Additionally, we examined the dose–response relationship between independent variables and CVD risk through the application of a four-knot restricted cubic spline (RCS) model. This approach allows for a detailed characterization of potential nonlinear associations while maintaining flexibility within the constraints defined by the spline knots. Furthermore, the diagnostic performance of AIP, NLR, and their combination, was assessed using receiver operating characteristic (ROC) curve analysis. This technique evaluates the trade-off between sensitivity and specificity across varying thresholds, with the area under the curve (AUC) serving as a robust metric to quantify discriminatory capacity. Higher AUC values reflect superior diagnostic accuracy, providing insights into the effectiveness of these biomarkers as predictors of CVD risk. All analyses were conducted using *R* (version 4.3.1). A *p* value of less than 0.05 was considered to indicate a statistically significant difference.

## 3. Results

**Figure 1:**
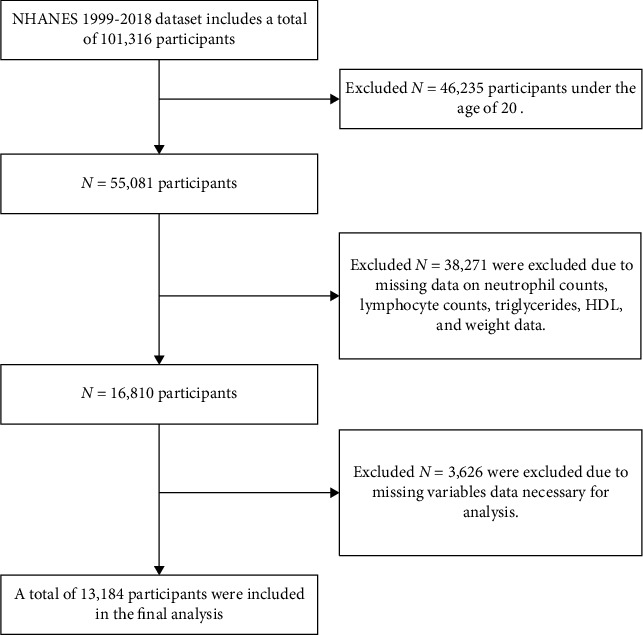
The flowchart of the study population selection process.

Our trial, conducted from 1999 to 2018, enrolled 101,316 participants. Excluding participants younger than 20 years old, missing key covariate results and missing basic variables observations, 13,184 individuals were included in the final analysis. The participant flow is depicted in Figure 1. We summarized the baseline clinical and biochemical characteristics of the participants based on their combined NLR and AIP grouping into four categories, as shown in Table 1. Group 1 includes participants with low NLR and low AIP levels, Group 2 includes those with high NLR and low AIP levels, Group 3 includes those with low NLR and high AIP levels, and Group 4 includes those with high NLR and high AIP levels. A higher proportion of participants aged over 65 years is observed in Group 4 (61.9%), and Female participants make up the majority in all groups. Regarding race, Non-Hispanic White participants were most represented, ranging from 67.2% in Group 1 to 73.1% in Group 3. Educational attainment revealed that most participants across all groups had some college or an associate degree, with percentages ranging between 34.7% in Group 1 and 40.2% in Group 2. Socioeconomic status, measured by the family income-to-poverty ratio, indicated that the majority fell within the range of 3.0 to 4.9 across all groups. Additionally, Participants in group 4 were more likely to be older, male, non-Hispanic White, nondrinker, and current smoker. They also tended to have elevated levels of TG, LDL-C, TC.

In Table 2, we utilized three logistic regression models to independently assess the combined impact of NLR and AIP levels on the risk of incident CVD. Group 1, characterized by low NLR and low AIP levels, was designated as the reference category (OR = 1.00). In Model I, which included no covariate adjustments, the OR for CVD risk in Group 4 (high NLR and high AIP) compared to Group 1 was 2.83 (95% CI: 2.25, 3.55; *p*  < 0.05). With partial covariate adjustments in Model II (age, gender, ethnicity, education, and family income-to-poverty ratio), the OR was reduced to 1.96 (95% CI: 1.53, 2.51; *p*  < 0.05). Finally, Model III, with full covariate adjustments (including BMI, WC, SBP and DBP, urine creatinine, FBG, HbA1c%, TC, TGs, HDL-C, LDL-C, diabetes history, hypertension history, and coronary heart disease history), revealed an OR of 1.41 (95% CI: 1.06, 1.87; *p*  < 0.05), indicating a persistent association.

**Figure 2:**
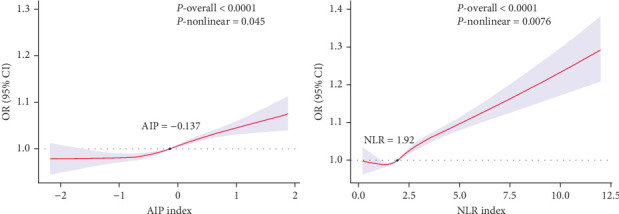
Adjusted dose-response relationship between AIP, NLR, and CVD. (A) AIP and CVD (reference point = −0.137). (B) NLR and CVD (reference point = 1.92). Assessed through RCS after adjusting for covariates. Adjusted covariates include sex, age, BMI, smoking status, diabetes, and hypertension. The solid red lines correspond to the central estimates, and the purple-shaded regions indicate the 95% confidence intervals.

In Table 3, the independent contributions of AIP and NLR to incident CVD risk were evaluated using logistic regression models. For AIP, quartile 1 (Q1) was designated as the reference category (OR = 1.00). The OR for CVD risk in Q4 compared to Q1 was 2.11 (95% CI: 1.74, 2.57; *p* ≤ 0.001) in Model I, 1.91 (95% CI: 1.55, 2.36; *p* ≤ 0.001) in Model II, and 1.54 (95% CI: 1.19, 1.98; *p* ≤ 0.001) in Model III.  Figure 2A depicts the RCS curve illustrating the dose–response relationship between AIP and CVD risk. The association is statistically significant, with a nonlinear relationship observed (*p*-overall < 0.0001; *p*-nonlinear = 0.045; reference point = −0.137). The curve shows a gradual increase in CVD risk at lower AIP values, followed by a sharp rise at higher AIP levels.

For NLR, quartile 1 (Q1) was also used as the reference category (OR = 1.00). The OR for CVD risk in Q4 compared to Q1 was 2.35 (95% CI: 1.89, 2.93; *p*  < 0.001) in Model I, 1.53 (95% CI: 1.22, 1.91; *p*  < 0.001) in Model II, and 1.54 (95% CI: 1.19, 1.98; *p*  < 0.001) in Model III.  Figure 2B illustrates the RCS curve for NLR, revealing a statistically significant nonlinear U-shaped relationship with CVD risk (*p*-overall < 0.0001; *p*-nonlinear = 0.0076; reference point = 1.92). The curve initially demonstrates an increase in CVD risk at lower NLR values, a decrease at moderate NLR levels, and a sharp rise at higher NLR values.

**Figure 3:**
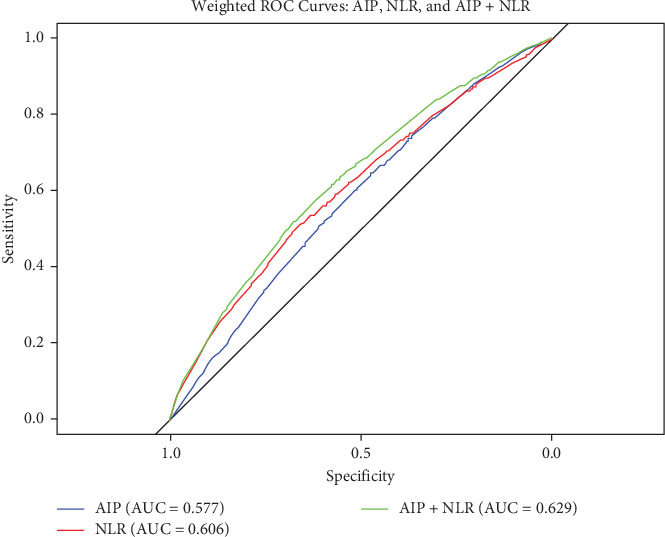
Receiver operating characteristic curves for NLR, AIP, and their combination for CVD.

**Figure 4:**
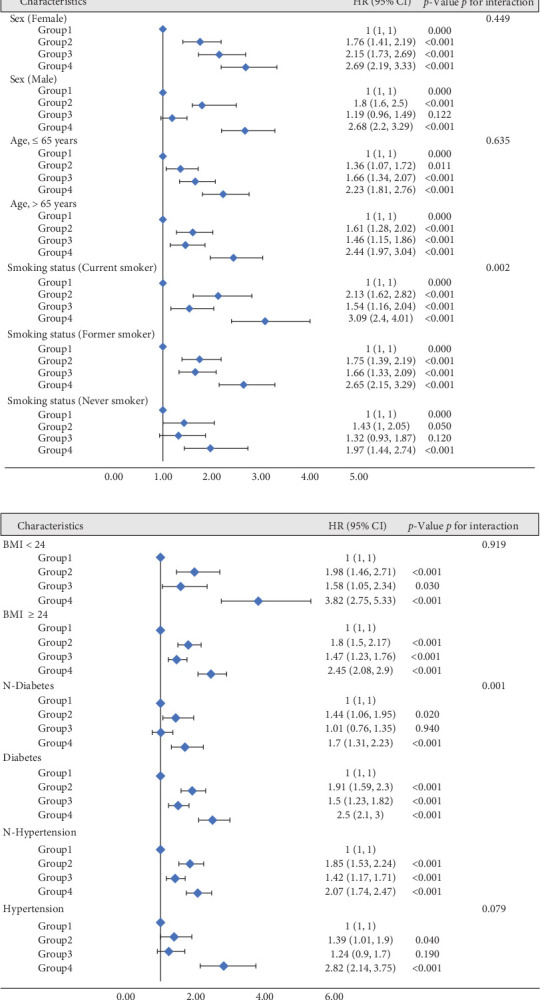
Subgroup analysis of the association between the change in NLR, AIP, and CVD risk. The reference group is Group 1, which represents participants with low NLR and low AIP levels. (A) Subgroups are sex, age, and smoking status. (B) Subgroups are BMI, diabetes, and hypertension.

Figure 3 presents the ROC curve analysis assessing the predictive performance of NLR, AIP, and their combination for CVD risk prediction. The AUC for AIP alone was 0.577 (95% CI: 0.563–0.594), and for NLR alone, it was 0.606 (95% CI: 0.589–0.621). When combining NLR and AIP into a single predictive metric, the AUC increased to 0.629 (95% CI: 0.615–0.646), indicating improved predictive efficacy compared to either NLR or AIP alone. Upon stratifying by sex, age, smoking, BMI, diabetes, and hypertension, we identified among participants aged >65 years, female, with BMI < 24 kg/m^2^, current smokers, diabetes, and hypertension, group 4 was linked with a heightened risk of CVD (*p* < 0.001) (Figure 4).

## 4. Discussion

This large-scale cohort study, conducted over nearly two decades (1999–2018), provides comprehensive insights into the association between the NLR, AIP, and their combined impact on CVD risk. Our results underscore the significant combined influence of elevated NLR and AIP levels (Group 4) on CVD risk. This persistent association emphasizes the synergistic effect of inflammatory and lipid-related pathways in driving cardiovascular risk. The combination of NLR and AIP showed superior predictive accuracy for CVD risk (AUC: 0.629) compared to AIP (AUC: 0.577) or NLR (AUC: 0.606) alone. Subgroup analysis identified heightened CVD risk in those aged >65, females, smokers, individuals with BMI < 24 kg/m^2^, diabetes, or hypertension when both markers were elevated. These results add to the growing body of evidence on inflammatory and lipid markers in CVD.

NLR is a biomarker that can be obtained by dividing neutrophil and lymphocyte counts by routine blood analysis [32]. In clinical practice, NLR is widely used because of its simplicity, low cost, and reliability of results [33]. Its main advantage is that NLR can effectively reveal the body's inflammatory response and immune status. Neutrophils, through the release of arachidonic acid, contribute to the inflammatory response, influencing blood coagulation and participating in pathways that regulate adaptive immune responses and chronic inflammation [34]. Inflammation is a pivotal factor in the pathogenesis and progression of CVD. The activation of long-term oxidative stress and systemic inflammation can result in endothelial damage, plaque rupture, and acute coronary events, thereby influencing the prognosis of CVD [35]. As the CANTOS study has further validated the inflammation hypothesis, providing direct evidence that monitoring inflammatory responses could be an effective therapeutic target for CVD [36]. Our results also show that the highest quartile of NLR (OR 1.54, 95% CI: 1.19–1.98) is significantly associated with CVD events compared to the lowest quartile of NLR.

Dysregulation of lipoprotein has been identified as the main pathological mechanism driving the instability of atherosclerotic plaques. A large number of studies have believed that TG, LDL-C, HDL-C and small-density low-density lipoprotein (sdLDL) are closely related to the incidence of CVD [37, 38]. In an ethnically based cohort study, sdLDL, apolipoprotein B, and LDL-C were found to be predictive of CVD risk in healthy adults with normal blood sugar levels [38]. In a study based on elderly and elderly Japanese people, it was observed that elevated sdLDL levels significantly increased the risk of CVD, regardless of serum LDL-C levels [39]. These results suggest that sdLDL appears to have a stronger predictive power than LDL-C in serum. Although the role of sdLDL in the development of CVD has been confirmed, its detection method is too complex and expensive to be widely implemented in clinical application [40]. Compared with sdLDL, AIP, as a comprehensive blood lipid indicator, can not only reflect sdLDL through the TG/HDL-C ratio but also reflect the size of LDL-C. It can more accurately reflect the patient's comprehensive lipid metabolism level than single blood lipid measurement and has a simpler way to obtain it [25]. The association between AIP and CVD has been substantiated by a plethora of studies. A retrospective study found that people with coronary artery stenosis showed higher AIP than people with coronary artery stenosis <50% [41]. An epidemiological study from South Korea showed a positive correlation between an increased risk of CVD and AIP levels [26], which is consistent with our findings. Our research also found that the highest quartile of AIP (OR 1.54, 95% CI: 1.19–1.98) is significantly associated with CVD events compared to the lowest quartile of AIP.

In recent years, a large number of studies have evaluated the relationship between individual NLR or AIP levels and CVD. However, no study has yet explored whether there have been previous synergistic benefits between the two to increase the ability to predict CVD incidence. Our results showed that participants with higher levels of both AIP and NLR had a significantly higher OR of 1.41 (1.06, 1.87) in terms of combined effects. The combination of NLR and AIP had a better predictive efficacy (AUC: 0.629) on CVD than alone. Based on this, we believed that NLR and AIP could serve as a useful, inexpensive, and accurate set of tools to predict CVD risks. As mentioned earlier, studies have analyzed the combined use of inflammatory indicators and lipid-related indicators. They have found that SII and TyG have the same trend in CVD, while the combination of TyG and NLR improves the accuracy of diagnosis of coronary artery disease [7, 12]. These studies all emphasize the importance of the use of composite indices in predicting CVD. TyG does an excellent job in capturing risks associated with insulin resistance [42], while SII reflects broader immune disorders [43]. NLR more reflects innate immunity or stress response, which can indicate that subclinical inflammation leads to endothelial damage [44, 45]. AIP is closely related to atherosclerosis, indicating that proatherogenic lipids penetrate the inflammatory vessel wall [46, 47]. Their synergistic effect may be more reliable for CVD prediction. Not only that, NLR and AIP are derived from routine complete blood counts and standard lipid testing, making them easy to use and cost-effective clinically.

The main advantage of our study is that it has a large sample and extended follow-up of CVD, providing more reliable conclusions. Our findings are also supported by the fact that we used the NHANES cohort, which was built and collected in a very tightly controlled manner, and that our large number of cohorts were adjusted for potential confounders, as well as detailed adjustments for subgroup analyses for primary outcomes. There are also limitations to our study. First, because the researchers are from the NHANES coronary heart disease population in the United States, this conclusion may not be generalized to other populations, and our future research will focus on multicenter and multi-international studies. Second, we are aware that there may be internal validity issues in the definition of self-reported CVD outcomes. Given the limitations of research resources and design, we chose self-reporting as the data collection method. To further enhance the reliability and validity of this study, we plan to collect more clinical diagnostic data in future research to supplement and verify the self-reported data. This method will help to assess the outcomes of CVDs more accurately and enhance the credibility of research findings. Third, although the combined predictive ability of NLR and AIP is statistically significant, the model with an AUC of 0.629 can only preliminarily identify high-risk subgroups in a wide population, but is insufficient to predict the levels of smaller individuals. Therefore, more populations with CVD characteristics need to be included and more diverse algorithm models need to be used to optimize the existing predictive ability. Finally, we adjusted for covariates that could have an effect when performing the logistic regression model. However, we could not rule out all potential residual confounders affecting NLR or AIP in observational studies. For example, the use of drugs (such as statins, antihypertensive drugs) may alter lipids or inflammatory markers. Different dietary structures may affect inflammation, lipid metabolism and cardiovascular health. Respondents may have acute inflammation or infection at the moment, which may temporarily increase NLR. The inability to fully explain these and other unmeasured confounding factors remains a major limitation. In the future, more complete evaluation indicators are needed to improve the assessment of these potential influencing factors so as to more comprehensively exclude the impact of confounding factors on study results.

## 5. Conclusion

Our research underscores the significant relationship between NLR, AIP, and CVD risk. The combined impact of NLR and AIP levels is strongly associated with CVD, emphasizing their relevance as markers in understanding the interplay between inflammation and lipid dysregulation in cardiovascular health. Measuring these indicators provides valuable insights into the underlying mechanisms of CVD and highlights the importance of managing inflammation and lipid profiles. To strengthen these findings, further high-quality, multicenter research is essential to validate and expand on this relationship.

## Figures and Tables

**Table 1:** Baseline characteristics of study participants.

Variable	Overall, *N* = 13,184 (100%)^a^	Group 1, *N* = 3481 (27%)^a^	Group 2, *N* = 3054 (24%)^a^	Group 3, *N* = 3253 (23%)^a^	Group 4, *N* = 3396 (26%)^a^	*p*-Value^b^
Age (years)	46.86 (16.73)	43.64 (16.39)	47.92 (17.54)	46.13 (16.08)	49.94 (16.24)	<0.001
Age group	—	—	—	—	—	<0.001
≤65 years	10,234 (84%)	2909 (89%)	2241 (81%)	2634 (87%)	2450 (81%)	—
>65 years	2950 (16%)	572 (11%)	813 (19%)	619 (13%)	946 (19%)	—
Sex	—	—	—	—	—	<0.001
Female	6681 (51%)	2066 (60%)	1751 (60%)	1387 (42%)	1477 (43%)	—
Male	6503 (49%)	1415 (40%)	1303 (40%)	1866 (58%)	1919 (57%)	—
Race	—	—	—	—	—	<0.001
Mexican American	2151 (7.9%)	448 (6.9%)	390 (6.1%)	682 (11%)	631 (8.1%)	—
Other Hispanic	1121 (4.7%)	260 (4.5%)	226 (3.9%)	336 (5.9%)	299 (4.4%)	—
Non-Hispanic White	6274 (71%)	1373 (65%)	1618 (74%)	1345 (66%)	1938 (77%)	—
Non-Hispanic Black	2538 (11%)	1078 (17%)	575 (9.8%)	594 (10%)	291 (4.5%)	—
Other/multiracial	1100 (6.4%)	322 (6.7%)	245 (5.8%)	296 (7.6%)	237 (5.5%)	—
Education attainment	—	—	—	—	—	<0.001
Less than 9th grade	1348 (5.2%)	252 (3.7%)	268 (4.6%)	417 (6.8%)	411 (6.1%)	—
9–11th grade	1932 (11%)	472 (9.5%)	389 (9.5%)	507 (12%)	564 (13%)	—
High school grade/GED	3040 (23%)	713 (20%)	684 (23%)	819 (24%)	824 (25%)	—
Some college or AA degree	3765 (31%)	1078 (32%)	872 (30%)	869 (30%)	946 (31%)	—
College graduate or above	3099 (30%)	966 (35%)	841 (33%)	641 (26%)	651 (24%)	—
Family income–poverty ratio	3.02 (1.62)	3.14 (1.63)	3.06 (1.60)	2.96 (1.64)	2.93 (1.60)	<0.001
Alcohol intake	—	—	—	—	—	<0.001
Nondrinker	6575 (50%)	1605 (47%)	1473 (49%)	1685 (52%)	1812 (53%)	—
1–5 drinks/month	1891 (17%)	584 (20%)	512 (20%)	380 (15%)	415 (15%)	—
5–10 drinks/month	1014 (9.4%)	287 (10%)	242 (8.9%)	258 (10%)	227 (8.3%)	—
10+ drinks/month	3703 (23%)	1004 (23%)	827 (22%)	930 (23%)	942 (24%)	—
Unclear	1 (<0.1%)	1 (<0.1%)	0 (0%)	0 (0%)	0 (0%)	—
Smoking status	—	—	—	—	—	<0.001
Never smoker	2726 (21%)	566 (15%)	606 (21%)	718 (23%)	836 (25%)	—
Former smoker	3384 (25%)	737 (23%)	780 (24%)	855 (26%)	1012 (29%)	—
Current smoker	7074 (54%)	2178 (62%)	1668 (55%)	1680 (51%)	1548 (46%)	—
BMI (kg/m^2^)	28.76 (6.63)	26.62 (5.88)	27.34 (6.52)	30.07 (6.07)	31.16 (6.89)	<0.001
WC (cm)	98.75 (16.31)	91.84 (14.36)	94.94 (15.71)	102.41 (14.63)	106.27 (16.18)	<0.001
Creatinine, urine (mg/dL)	125.89 (75.81)	120.56 (77.43)	118.47 (73.62)	134.00 (78.01)	131.02 (72.93)	<0.001
TC (mmol/L)	5.01 (1.04)	4.88 (0.98)	4.82 (0.98)	5.25 (1.08)	5.10 (1.06)	<0.001
HDL-C (mmol/L)	1.41 (0.42)	1.65 (0.39)	1.64 (0.42)	1.18 (0.26)	1.16 (0.26)	<0.001
TG (mmol/L)	1.36 (0.76)	0.83 (0.27)	0.86 (0.28)	1.91 (0.73)	1.90 (0.72)	<0.001
LDL-C (mmol/L)	2.97 (0.91)	2.85 (0.83)	2.79 (0.84)	3.20 (0.96)	3.06 (0.93)	<0.001
Diabetes, *N* (%)	—	—	—	—	—	<0.001
Yes	2199 (12%)	328 (6.0%)	401 (9.5%)	648 (14%)	822 (20%)	—
No	10,985 (88%)	3153 (94%)	2653 (90%)	2605 (86%)	2574 (80%)	—
FPG, mmol/L	5.77 (1.51)	5.43 (1.02)	5.59 (1.18)	5.94 (1.66)	6.16 (1.90)	<0.001
HbA1c (%)	5.56 (0.87)	5.40 (0.66)	5.46 (0.68)	5.67 (0.99)	5.74 (1.05)	<0.001
Hypertension	—	—	—	—	—	<0.001
Yes	5514 (37%)	1169 (27%)	1209 (34%)	1414 (38%)	1722 (49%)	—
No	7670 (63%)	2312 (73%)	1845 (66%)	1839 (62%)	1674 (51%)	—
SBP (mmHg)	121.15 (16.84)	118.36 (16.62)	119.83 (17.32)	122.35 (15.70)	124.24 (17.02)	<0.001
DBP (mmHg)	69.39 (12.09)	68.44 (11.29)	67.71 (12.06)	70.81 (11.82)	70.66 (12.88)	<0.001
Platelet, × 10^9^/L	248.57 (65.45)	240.30 (60.55)	248.90 (66.82)	252.90 (64.60)	253.10 (69.02)	<0.001
Neutrophils, × 10^9^/L	4.00 (1.63)	2.93 (0.89)	4.63 (1.62)	3.44 (1.03)	5.07 (1.76)	<0.001
Lymphocyte, × 10^9^/L	2.00 (1.12)	2.11 (1.20)	1.64 (0.48)	2.45 (1.67)	1.82 (0.52)	<0.001
NLR	2.18 (1.12)	1.43 (0.34)	2.96 (1.25)	1.47 (0.34)	2.89 (1.03)	<0.001
AIP	−0.14 (0.71)	−0.71 (0.40)	−0.68 (0.42)	0.44 (0.42)	0.45 (0.42)	<0.001

*Note:* HbA1c%, glycated hemoglobin.

Abbreviations: AIP, atherogenic index of plasma; BMI, body mass index; DBP, diastolic blood pressure; FBG, fasting blood glucose; HDL-C, high-density lipoprotein cholesterol; LDL-C, low-density lipoprotein cholesterol; NLR, neutrophil-to-lymphocyte ratio; SBP, systolic blood pressure; TC, total cholesterol; TG, triglycerides; WC, waist circumference.

^a^ *N* (unweighted); *n* (weighted) (%).

^b^Design-based Kruskal Wallis test; Pearson's *X*^2^: Rao and Scott adjustment.

**Table 2:** The combined impact of AIP and NLR on the odds ratio of incident CVD risk among participants.

Characteristic	Model I			Model II			Model III		
	OR	95% CI	*p*-Value	OR	95% CI	*p*-Value	OR	95% CI	*p*-Value
Group 1	1.0 (Reference)	—	—	1.0 (Reference)	—	—	1.0 (Reference)	—	—
Group 2	1.78	1.45, 2.18	<0.001	1.27	1.03, 1.58	0.027	1.19	0.92, 1.53	0.2
Group 3	1.78	1.42, 2.23	<0.001	1.57	1.23, 2.02	<0.001	1.35	1.03, 1.79	0.032
Group 4	2.83	2.25, 3.55	<0.001	1.96	1.53, 2.51	<0.001	1.41	1.06, 1.87	0.018

*Note:* Model I was not adjusted for any other covariates. Model II was adjusted for age, sex, race, educational attainment, and family income–poverty ratio. Model III was adjusted for age, sex, race,educational attainment, and family income–poverty ratio, alcohol intake, smoking status, BMI, WC, urine creatinine, LDL-C, history of diabetes, history of hypertension, FBG, HbA1c%, SBP, DBP, Platelet. HbA1c%, glycated hemoglobin.

Abbreviations: BMI, body mass index; CI, confidence Interval; CVD, cardiovascular disease; DBP, diastolic blood pressure; FBG, fasting blood glucose; HDL-C, high-density lipoprotein cholesterol; LDL-C, low-density lipoprotein cholesterol; NLR, neutrophil-to-lymphocyte ratio; SBP, systolic blood pressure; TC, total cholesterol; TG, triglycerides; WC, waist circumference.

**Table 3:** The independent contributions of AIP and NLR to the risk of incident CVD among participants.

Characteristic	Model I			Model II			Model III		
	OR	95% CI	*p*-Value	OR	95% CI	*p*-Value	OR	95% CI	*p*-Value
AIP (continuous)	1.42	1.30, 1.54	<0.001	1.4	1.26, 1.55	<0.001	1.23	1.08, 1.41	0.002
Q1	1.0 (Reference)	—	—	1.0 (Reference)	—	—	1.0 (Reference)	—	—
Q2	1.24	1.01, 1.52	0.036	1.12	0.92, 1.37	0.3	1.1	0.86, 1.40	0.4
Q3	1.69	1.38, 2.07	<0.001	1.41	1.14, 1.74	0.002	1.14	0.89, 1.46	0.3
Q4	2.11	1.74, 2.57	<0.001	1.91	1.55, 2.36	<0.001	1.54	1.19, 1.98	0.001
NLR (continuous)	1.29	1.22, 1.36	<0.001	1.11	1.06, 1.17	<0.001	1.07	1.01, 1.14	0.028
Q1	1.0 (Reference)	—	—	1.0 (Reference)	—	—	1.0 (Reference)	—	—
Q2	1.23	0.98, 1.55	0.074	1.22	0.96, 1.55	0.1	1.09	0.82, 1.44	0.6
Q3	1.47	1.17, 1.84	0.001	1.27	1.00, 1.61	0.053	1.04	0.78, 1.38	0.8
Q4	2.35	1.89, 2.93	<0.001	1.53	1.22, 1.91	<0.001	1.25	0.97, 1.62	0.088

*Note:* Model I was not adjusted for any other covariates. Model II was adjusted for age, sex, race, educational attainment, and family income–poverty ratio. Model III was adjusted for age, sex, race,educational attainment, and family income–poverty ratio, alcohol intake, smoking status, BMI, WC, urine creatinine, LDL-C, history of diabetes, history of hypertension, FBG, HbA1c%, SBP, DBP, Platelet. HbA1c%, glycated hemoglobin.

Abbreviations: BMI, body mass index; CI, confidence Interval; CVD, cardiovascular disease; DBP, diastolic blood pressure; FBG, fasting blood glucose; HDL-C, high-density lipoprotein cholesterol; LDL-C, low-density lipoprotein cholesterol; NLR, neutrophil-to-lymphocyte ratio; SBP, systolic blood pressure; TC, total cholesterol; TG, triglycerides; WC, waist circumference.

## Data Availability

The datasets used and/or analyzed during the current study were publicly available. These data can be found at https://www.cdc.gov/nchs/nhanes/index.htm.

## Nomenclature

AIP: Atherogenic index of plasma
BMI: Body mass index
CI: Confidence interval
CVD: Cardiovascular disease
DBP: Diastolic blood pressure
FBG: Fasting blood glucose
HbA1c%: Glycated hemoglobin
HDL-C: High-density lipoprotein cholesterol
LDL: Low-density lipoprotein
LDL-C: Low-density lipoprotein cholesterol
LMR: Lymphocyte-to-monocyte ratio
NLR: Neutrophil-to-lymphocyte Ratio
OR: Odds ratio
RCS: Restricted cubic spline
SBP: Systolic blood pressure
SII: Systemic immune-inflammation index
TC: Total cholesterol
TG: Triglycerides
TyG: Triglyceride-glucose
WC: Waist circumference.
